# Cervical Cancer Diagnosis Using an Integrated System of Principal Component Analysis, Genetic Algorithm, and Multilayer Perceptron

**DOI:** 10.3390/healthcare10102002

**Published:** 2022-10-11

**Authors:** Odai Y. Dweekat, Sarah S. Lam

**Affiliations:** Department of Systems Science and Industrial Engineering, Binghamton University, Binghamton, NY 13902, USA

**Keywords:** cervical cancer, genetic algorithm, machine learning, multilayer perceptron, principal component analysis

## Abstract

Cervical cancer is one of the most dangerous diseases that affect women worldwide. The diagnosis of cervical cancer is challenging, costly, and time-consuming. Existing literature has focused on traditional machine learning techniques and deep learning to identify and predict cervical cancer. This research proposes an integrated system of Genetic Algorithm (GA), Multilayer Perceptron (MLP), and Principal Component Analysis (PCA) that accurately predicts cervical cancer. GA is used to optimize the MLP hyperparameters, and the MLPs act as simulators within the GA to provide the prediction accuracy of the solutions. The proposed method uses PCA to transform the available factors; the transformed features are subsequently used as inputs to the MLP for model training. To contrast with the PCA method, different subsets of the original factors are selected. The performance of the integrated system of PCA–GA–MLP is compared with nine different classification algorithms. The results indicate that the proposed method outperforms the studied classification algorithms. The PCA–GA–MLP model achieves the best accuracy in diagnosing Hinselmann, Biopsy, and Cytology when compared to existing approaches in the literature that were implemented on the same dataset. This study introduces a robust tool that allows medical teams to predict cervical cancer in its early stage.

## 1. Introduction

Cancer is a leading cause of death across the world. In 2020, around 604,000 women were diagnosed with cervical cancer and 342,000 cervical cancer deaths were recorded [1]. Cervical cancer is one of the most dangerous diseases for women, and approximately 80% of those diagnosed were aged 15 to 45.

Cervical cancer is caused by mutations in genes that regulate cell division and proliferation. Two associated symptoms of early-stage cervical cancer are pelvic discomfort and vaginal bleeding. Because cervical cancer cannot be diagnosed in its early stages, these symptoms are the only early warning signs. If gone unnoticed, cervical cancer can spread to other body regions, such as the lungs and abdomen. Cervical cancer can be identified with diffusion-weighted and Magnetic Resonance Imaging in its later stages (diffusion-weighted imaging). Symptoms, which include tiredness, back discomfort, leg pain, weight loss, and potential bone fractures, often become more severe as the disease progresses [1].

Cervical cancer risk is raised by early pregnancy, contraception usage, numerous pregnancies, cigarette use, and Human Papillomavirus (HPV) [2]. HPV, one of the most critical risk factors for cervical cancer, is a DNA virus that spreads mainly through sexual interaction. HPV is identified in cervical cancer patients 99.7% of the time and consists of more than 100 distinct factors [3].

Cervical cancer is detected using four specific tests: Hinselmann, Schiller, Cytology, and Biopsy (HSCB). Hinselmann is a member of the “colposcopy with acetic acid” group. Schiller, Cytology, and Biopsy, on the other hand, use Lugol iodine [4]. Diagnosing cervical cancer is expensive and time-consuming. Unfortunately, low-income nations face significant challenges in raising cancer awareness and screening. In addition, a lack of resources, such as medical expertise, equipment, and specialist doctors, contributes to the spread of cervical cancer in developing nations. As a result, patient fatality rates are rising [5].

In recent decades, Machine Learning (ML) techniques have been used to identify and predict cervical cancer. Among the most widely used approaches are Neural Network (NN), Ensemble Learning, Support Vector Machine (SVM), K-Nearest Neighbor (KNN), Texture Analysis, and Deep Learning [6,7,8,9,10,11,12,13,14,15,16,17].

One of the most effective techniques for predicting cervical cancer is NN. There are several types of NNs, such as Multilayer Perceptron (MLP), Convolution Neural Network (CNN), Probabilistic Neural Network (PNN), and Recurrent Neural Network (RNN) [18,19]. Many researchers over the last decade have endorsed the use of MLP when predicting cervical cancer because it provides a decent classification accuracy [19,20].

**Figure 1:**
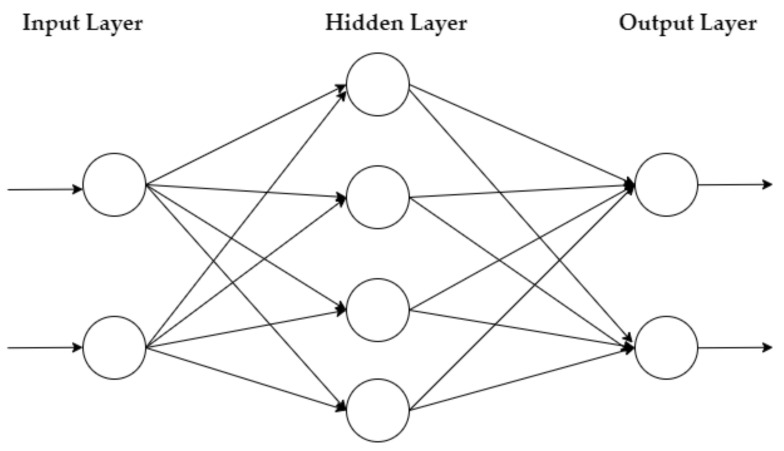
MLP architecture.

The MLP is a feedforward NN that learns via the Backpropagation method. It has an input layer of neurons that function as receivers, and it contains one or more hidden layers of neurons that compute data and iterate. Finally, an output layer predicts the result, as summarized in Figure 1.

Genetic Algorithm (GA) is a stochastic population searching technique that examines large search areas efficiently. GA has been used to optimize a range of MLP parameters, including momentum, size of the neurons, stopping criteria, solver(s), and activation function(s) [19,21].

This paper is organized as follows: Section 2 discusses related literature on cervical cancer prediction. Section 3 summarizes the contribution of this research. Section 4 describes the research methods, data preprocessing, dataset for the study, and procedures used to combine GA with MLP. Section 5 discusses the results of the proposed method and compares the proposed method with other classification approaches. Finally, Section 6 provides the conclusions and future research directions.

## 2. Related Literature

This section discusses and criticizes relevant current ML approaches for predicting cervical cancer. Most of the studies that have been conducted to predict cervical cancer used the cervical cancer dataset from the University of California Irvine (UCI) Machine Learning Repository.

Wu and Zhou [4] implemented three different methods to diagnose four target variables of cervical cancer: HSCB. The dataset contained 32 factors that potentially cause cervical cancer. The authors used the Random Oversampling method to balance the dataset of 668 patients. They combined SVM with Recursive Feature Elimination (RFE), combined SVM with Principal Component Analysis (PCA), and employed the traditional SVM, and they compared these method’s characterization of cervical cancer. The classification results show that SVM–RFE and SVM–PCA provide more accurate results when selecting only eight factors instead of using the traditional SVM techniques [4,22].

Abdoh et al. [22] predicted cervical cancer by using RFE and PCA to eliminate several features. The researchers used the Synthetic Minority Oversampling Technique (SMOTE) to balance the cervical cancer dataset from UCI. Random Forest (RF) was used to diagnose cervical cancer and was compared with RF–PCA and RF–RFE. Their results show that integrating SMOTE with RF classifiers enhances classification accuracy by about 4% when compared with the work conducted by Wu and Zhou [4].

Deng et al. [23] applied RF, SVM, and eXtreme Gradient Boosting (XGBoost) to diagnose four target variables of cervical cancer: HSCB. Similar to Abdoh et al. [22], the authors used SMOTE to balance the dataset. However, their research did not use feature elimination techniques. Their results show that RF and XGBoost performed better than SVM in terms of classification accuracy. The results of Deng et al. [23] are similar to those reported by Abdoh et al. [22].

Adem et al. [15] used Deep Learning to predict the same dataset of cervical cancer by employing Softmax classification with a stacked autoencoder. The stacked autoencoder was used as a dimensional reduction tool, while the softmax layer was used to predict HSCB of cervical cancer. The authors validated their approach with six ML methods: RF, Decision Tree (DT), MLP, SVM, Rotation Forest models, and KNN [15]. Their proposed approach for diagnosing the four target variables (HSCB) of cervical cancer achieved better performance than the method of Wu and Zhou (2017), with close to 4% improvement in classification accuracy.

Alsmariy et al. [24] used RF, Logistic Regression (LR), and DT to diagnose HSCB of cervical cancer. Similar to Deng et al. [23] and Abdoh et al. [22], the authors applied SMOTE to balance the dataset. They used PCA as a feature reduction approach. They implemented a voting technique that enables several algorithms to vote to select a winner. Their results perform better than those of Abdoh et al. [22], Deng et al. [23], and Wu and Zhou [4].

Wahid and Al-Mazini [5] adopted a meta-heuristic algorithm, Ant Colony Optimization (ACO) with the Ant-Miner data classification rule, to select the most critical risk factors to diagnose HSCB of cervical cancer. The authors claimed that their method was used for the first time in the literature for the UCI cervical cancer dataset. Their classification results perform better than those of Wu and Zhou [4] but are inferior to the reported results of Alsmariy et al. [24], Abdoh et al. [22], and Deng et al. [23].

Other researchers such as Devi et al. [10] and Fernandes et al. [14] used image processing and Deep Learning to diagnose and predict cervical cancer with a different dataset from the UCI. Devi et al. [10] classified cervical cancer into normal and abnormal cells using NN and Learning Vector Quantification (LVQ). Digital photographs of patients were used as inputs. To diagnose cervical cancer, LVQ was used to obtain the coefficient mean value of the extracted photographs. Their model achieves a 90% classification accuracy [10].

In contrast, deep learning algorithms were used by Fernandes et al. [14] to predict cervical cancer. Their approach is founded on a loss function that permits dimensional reduction to identify the most important classification variables. They concentrate on a specific form of cervical cancer called biopsy. Their algorithm achieves an Area Under the Curve (AUC) of 0.6875 [14].

There were a few studies that combined GA with MLP to diagnose cervical cancer. In one study, GA was used to determine the optimal initial weights and bias of MLP to classify cervical cancer [25]. The study was conducted on a dataset with 401 patients, of which 51.2% had cervical cancer, and included 16 risk factors. The accuracy of their proposed model improved from 94.51% to 96.26% when combining GA with MLP. Similarly, another study adopted GA to optimize the MLP’s initial weights and threshold to identify Nanoparticle (NP) sensors in the early diagnosis of cervical cancer cells. Their study compared the performance of the GA and MLP combination with a standalone MLP. Their results indicated that combining GA with MLP achieved statistically better root mean Square (RMS) and Mean Absolute Error (MAE) than MLP alone [26].

In summary, most of the research used classical machine learning classification algorithms and deep learning approaches to diagnose cervical cancer. The literature on diagnosing HSCB cited above is also summarized in Section 5 where it compared with the proposed hybrid system of PCA–GA–MLP. There were two studies that used GA to optimize MLP’s initial weights, threshold, or bias to diagnose cervical cancer. However, none of the research optimized the parameters of MLP, which include the size of each hidden layer, solvers, and activation functions, to diagnose cervical cancer. Further, a hybrid model of PCA–GA–MLP for the diagnosis of cervical cancer has not been proposed in the literature.

## 3. Contribution

This study is the first that integrates PCA, GA, and MLP altogether in one framework that accurately predicts cervical cancer using the benchmark dataset from UCI. The proposed method transforms all available features using the PCA method. The transformed features are utilized in model constructions of the MLP, which is within a hybrid system of GA and MLP. GA is used to optimize the MLP parameters, whereas the MLP acts as a simulator within the GA. The hybrid system iteratively evolves the optimal design of MLP that provides the best cervical cancer classification accuracy. The developed framework introduces a robust tool that allows medical teams to predict cervical cancer as a preventive strategy that reduces cervical cancer rates and costs while improving the quality of care for cancer patients.

## 4. Research Methodology

**Figure 2:**
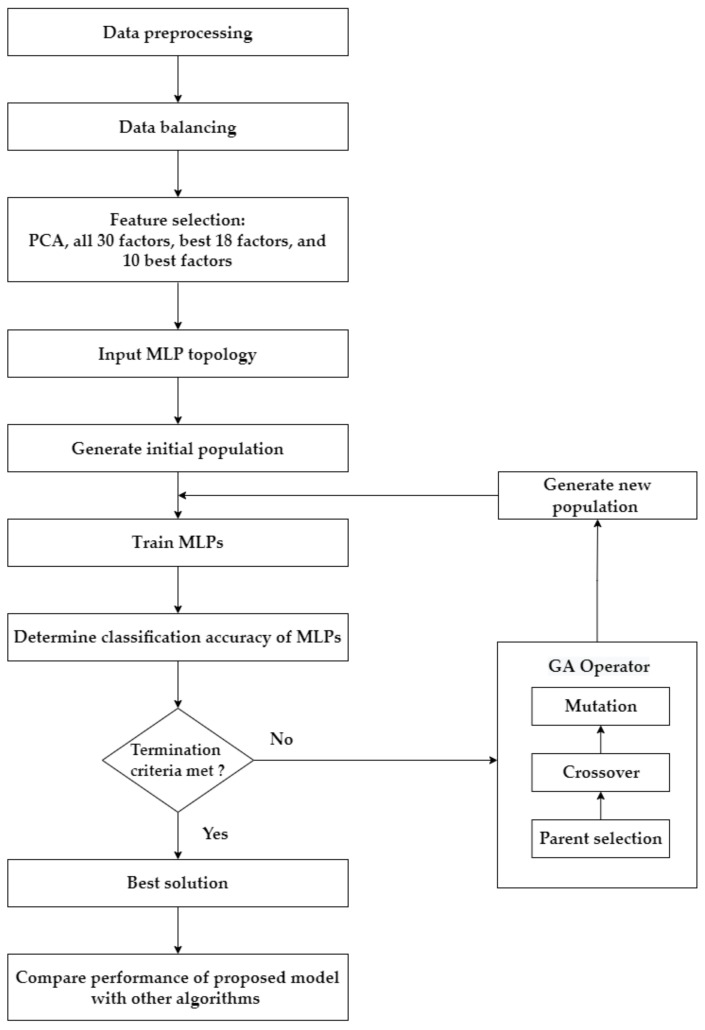
The framework of the proposed approach.

This research has four main steps, as summarized in Figure 2. Step 1 involves preprocessing and balancing the dataset. Step 2 describes the application of the feature selection process. In this research, four feature selection approaches separately diagnose each target variable/test of cervical cancer: using the transformed features from PCA; using all original features; using the top 18 features based on RF importance; and using the top 10 features based on RF importance. Step 3 explains how GA is used as an optimization tool to determine the optimal parameters of the MLP to predict cervical cancer. This process is applied to the four target variables (HSCB) of cervical cancer separately. Four feature selection approaches are implemented for each target variable, which results in 16 different scenarios (i.e., four scenarios for each cervical cancer variable: PCA–GA–MLP, GA–MLP using all 30 factors, GA–MLP using the top 18 factors, and GA–MLP using the top 10 factors). Step 4 determines the performance measures for each scenario using a 5-fold cross-validation and compares each scenario’s results with nine other classification algorithms. Five-fold cross-validation is used because it allows all available data instances to be used for both model development and model validation [27]. The nine algorithms are as follows: RF, Linear Discriminant Analysis (LDA), SVM, LR, Gaussian Naïve Bayes (NB), KNN, DT, Adaptive Boosting (AdaBoost), and Centroid-Displacement-based KNN (CD-KNN) [28]. Therefore, a total of 160 different experiments are implemented.

Over the past decade, researchers have advocated the use of MLP in cervical cancer prediction due to its respectable classification accuracy [10,18,19,20,25,26,29,30]; therefore, MLP is selected for optimization by GA in this study. In addition, various researchers used SVM, RF, LR DT, KNN, NB, LDA, and AdaBoost to classify cervical cancer [4,9,10,11,13,22,23,30,31,32,33]. Those algorithms are among the most common classification algorithms in modeling medical datasets. Therefore, those algorithms are used in this study to validate the proposed approach, and their results are compared. 

### 4.1. Dataset Description and Data Preprocessing

UCI published the cervical cancer dataset. The dataset contains 858 patients that have 32 cervical cancer factors as summarized in Table 1. It has four cervical cancer target variables: Hinselmann, Schiller, Cytology, and Biopsy. Each cervical cancer target variable is treated as a separate problem in this research. Therefore, four datasets are prepared to be used separately: Hinselmann, Schiller, Cytology, and Biopsy.

Because some patients refused to answer personal questions, some data are missing. As a result, the dataset needed to be preprocessed to account for the null data. Two factors were removed due to having a large amount of missing data (i.e., factor numbers 27, and 28 in Table 1). Further, some samples were removed for the same reason. As a result, there are 668 patients in the final (preprocessed) dataset. Each data instance has 30 distinct risk factors and four target variables (HSCB) of cervical cancer. Normalization is used for some numerical factors to eliminate data redundancy and avoid any undesirable characteristics due to the wide range of values in those factors. The percentage of cervical cancer patients within the dataset is 4.5% for Hinselmann, 9.4% for Schiller, 5.8% for Cytology, and 6.7% for Biopsy. Therefore, the random oversampling technique is used to balance the unbalanced dataset.

### 4.2. Feature Selection and Principal Component Analysis

Feature selection is the process of minimizing the number of input variables when creating a predictive model. The number of input variables might be reduced to decrease the computational cost of modeling and, in some circumstances, to improve the model’s performance [33,34].

RF is a Bagging algorithm that mixes several different DTs. Tree-based techniques in RFs are naturally rated by how well they improve node purity or, in other words, how well they minimize impurity (Gini impurity) across all trees. The nodes with the greatest reduction in impurity are located at the beginning of the trees, while those with the smallest reduction are found at the conclusion. A subset of the most important features may be created by pruning trees below a certain node [35,36,37]. The proposed framework investigates two feature selection methods, PCA and RF importance, where RF is used to select the best 18 factors and the best 10 factors with the highest relative importance in predicting cervical cancer, as shown in Table 2 and Table 3. The factors listed in Table 2 and Table 3 refer to the factor numbers provided in Table 1.

PCA is a statistical method that uses the eigenvector to determine the orientation of features. PCA’s fundamental idea is to map a j-dimensional feature space into an i-dimensional space, which is generally known as the principal components, where i < j. The covariance matrix is calculated, and the eigenvectors and eigenvalues are computed. Because an eigenvalue shows the most significant relationship between the dataset characteristics, the eigenvector with the greatest eigenvalue is selected as the principal component of the cervical cancer dataset. The eigenvalues are sorted in ascending order to select the most significant principal component(s), while the lowest eigenvalues are discarded. This process reduces large dimensional datasets to smaller dimensional datasets. The variance measures the dispersion of the data in the cervical cancer dataset. Lastly, eigenvectors and eigenvalues for the covariance matrix are computed. Eigenvalues are transformed using varimax orthogonal rotation or oblique rotation [38].

In this research, PCA transforms the 30 original factors into a two-dimensional space, which accounts for most of the variance in the original dataset. The PCA approach reduces the noise in the cervical cancer dataset and also reduces the computational time for model development.

### 4.3. Combination of GA–MLP

GA is used in this research as an optimization tool to determine the optimal hyperparameters for the MLP that provides the highest classification accuracy in diagnosing each target variable of cervical cancer. The MLP has several hyperparameters that need to be fine-tuned, which include the size of each hidden layer, solvers, and activation functions. The hyperparameters of MLP are encoded as chromosomes in the GA. The population of solutions/chromosomes in the GA represents a population of MLPs. The classification accuracy of each MLP, after network training is completed, is used as the fitness value of that solution.

Initially, the GA has a random population of solutions. In each generation, each solution (MLP) goes through the training process to determine its fitness value (classification accuracy). The evaluated solutions will then go through the typical evolution process of the GA: select two parent solutions, crossover the parent solutions to create two children with a probability of Pc, and mutate the children with a probability of Pm. Once the internal loop is completed and reaches half of the population size (n/2), the replacement process will then cull all the parent solutions, the children will advance to the next generation, and the generation counter will increase by one. This process will continue until the termination criteria are met (gmax). Finally, the best solution out of all generations will be selected, which represents the MLP’s optimized parameters for predicting patients with cervical cancer. Algorithm 1 represents the pseudocode of the hybrid GA–MLP for optimizing the MLP parameters.
**Algorithm 1.** Hybrid GA–MLP for optimizing MLP parameters1: Set GA parameters (Pc, Pm, n, gmax)2: Encode solutions (MLP parameters) using real value encoding3: Randomly generate n solutions4: Calculate the fitness value of each solution by the trained MLPs5: for i = 1, until gmax do6:   for i = 1, until n/2 do7:     Select two parents8:     Crossover to create two children with Pc9:     Mutate children with Pm10:   end for 11: Replace parents with children12: end for13: Return the best solution

### 4.4. Main Operators and MLP Hyperparameters

The following parameters are used to design the GA: tournament selection is used to select the parents (k = 4) for breeding; the crossover probability is set at 1 to perform a single-point crossover operation; the mutation probability is set at 0.001; the population size is fixed at 50; and the stopping criterion is 200 generations.

The hyperparameters of MLP are the encoded solution vectors in the GA. Each solution vector considers 4 types of activation functions, 3 types of solvers, and 50 different sizes for the first and the second layers in the MLP. However, some hyperparameters for the MLP are fixed: the learning rate is set at 0.001, the momentum is set at 0.90, and the stopping criterion is set to 200 iterations. Each solution undergoes network training, and the classification accuracy of the trained MLP is used as its fitness value in the GA. The proposed methodology is coded in the Python 3.9 environment.

### 4.5. Performance Metrics

Accuracy, sensitivity, specificity, and precision are the most common data mining performance metrics [39]. These performance metrics are defined in Equations (1)–(5). The confusion matrix output determines the following metrics: True Negative (TN), True Positive (TP), False Negative (FN), and False Positive (FP) [40]. TP is the number of correct predictions that a patient has cervical cancer, i.e., cervical cancer is correctly classified as cervical cancer. TN is the number of correct predictions that a patient does not have cervical cancer, i.e., non-cervical cancer is correctly classified as non-cervical cancer. FN is the number of incorrect predictions that a patient does not have cervical cancer, i.e., cervical cancer is identified as non-cervical cancer. FP is the number of incorrect predictions that a patient has cervical cancer, i.e., non-cervical cancer is detected as cervical cancer. Table 4 summarizes the confusion matrix for diagnosing cervical cancer.

(1) $$\mathrm{Accuracy}=\frac{\mathrm{TP}+\mathrm{TN}}{\mathrm{TP}+\mathrm{FP}+\mathrm{TN}+\mathrm{FN}}\;\times \;100$$

(2) $$\mathrm{Sensitivity}=\frac{\mathrm{TP}}{\mathrm{TP}+\mathrm{FN}}\;\times \;100$$

(3) $$\mathrm{Specificity}=\frac{\mathrm{TN}}{\mathrm{TN}+\mathrm{FP}}\;\times \;100$$

(4) $$\mathrm{Precision}=\frac{\mathrm{TP}}{\mathrm{TP}+\mathrm{FP}}\;\times \;100$$

(5) $$F1-\mathrm{Score}=\frac{2\times \;\mathrm{Sensitivity}\;\times \;\mathrm{Precision}\;}{\;\mathrm{Sensitivity}\;+\;\mathrm{Precision}}\;\times \;100$$

In cervical cancer, the performance metrics are interpreted as follows: accuracy refers to how well the model can accurately categorize TP and TN cervical cancer cases out of all instances. Sensitivity refers to the percentage that the model correctly classifies TP cervical cancer cases out of all patients with cervical cancer. The model’s specificity measures how well it can categorize individuals as not having cervical cancer out of those diagnosed without the disease. Precision refers to the percentage that the model correctly classifies TP cervical cancer cases out of all cases that are classified as cervical cancer. Finally, F1-score is generally considered a better performance measure; it represents a weighted average between sensitivity and precision.

## 5. Results and Discussion

Experimental results of each target variable of cervical cancer are discussed separately in this section. The proposed approach is compared with nine different classification algorithms. Further, the performance of the PCA–GA–MLP model is compared with the best results in the studies that were conducted on the same cervical cancer dataset from UCI.

### 5.1. Target Variable: Hinselmann

Table 5 summarizes the performance measures obtained when comparing GA–MLP with other classifiers to diagnose Hinselmann. When adopting PCA as a dimensional reduction tool, PCA–GA–MLP outperforms all nine classification algorithms; it has an accuracy of 98.20%, a sensitivity of 100.00%, a specificity of 96.37%, a precision of 96.54%, and an F1-score of 98.24%. PCA–AdaBoost is the next best method; it has an accuracy of 94.67%, a sensitivity of 100.00%, a specificity of 89.29%, a precision of 90.41%, and an F1-score of 94.96%.

When using all (available) 30 factors to diagnose Hinselmann, GA–MLP has the best accuracy, sensitivity, specificity, precision, and F1-score (97.57%, 100.00%, 95.15%, 95.36%, and 97.62%, respectively), followed by AdaBoost, KNN, and CD-KNN. When incorporating the top 18 factors to diagnose Hinselmann, GA–MLP provides the best results across all performance measures. When selecting the top 10 factors to diagnose Hinselmann, GA–MLP achieves the best accuracy, specificity, precision, and F1-score.

**Figure 3:**
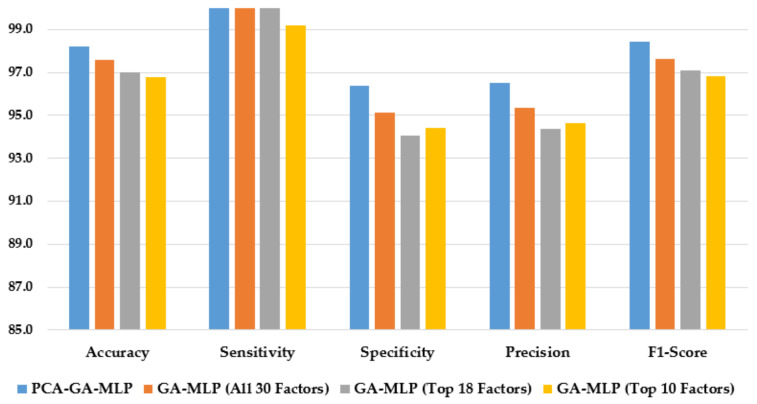
Comparison of the proposed method using four feature selection methods to diagnose Hinselmann.

Figure 3 illustrates the performance of the hybrid system of PCA–GA–MLP and the three GA–MLPs that used the top 10 factors, the top 18 factors, and all 30 factors to diagnose Hinselmann. The figure indicates that marginal improvement in model performance is observed when a larger number of factors is used in GA–MLP. Furthermore, the hybrid system of PCA–GA–MLP outperforms all three GA–MLPs across all performance measures. The PCA–GA–MLP offers a simpler input structure in the MLP as a result of the dimension reduction in PCA.

As shown in Table 6, PCA–GA–MLP performs better than the methods reported by Wu and Zhou [4], Adem et al. [15], Alsmariy et al. [24], Abdoh et al. [22], Deng et al. [23], and Wahid and Al-Mazini [5], in terms of accuracy, sensitivity, and F1-score. In terms of specificity, PCA–GA–MLP performs better than the method reported by Wu and Zhou [4].

### 5.2. Target Variable: Schiller

The performance measures of GA–MLP and other algorithms in diagnosing Schiller are summarized in Table 7. When applying PCA as a feature selection method, PCA–GA–MLP achieves the highest accuracy, sensitivity, specificity, precision, and F1-score of 96.78%, 100.00%, 93.61%, 93.97%, and 96.87%, respectively. PCA–CD-KNN is the second-best method, with an accuracy of 87.52%, a sensitivity of 99.15%, a specificity of 75.88%, a precision of 80.50%, and an F1-score of 88.83%.

When using all 30 factors to diagnose Schiller, GA–MLP has the best accuracy, precision, and F1-score (94.13%, 89.82%, and 94.45%, respectively), followed by CD-KNN and KNN. When using the best 18 factors to diagnose Schiller, GA–MLP provides the best results across all five performance measures. In selecting the best 10 factors to diagnose Schiller, GA–MLP performs better than all other classifiers, in terms of accuracy, sensitivity, precision, and F1-score (93.39%, 99.17%, 88.98%, and 93.79%, respectively).

**Figure 4:**
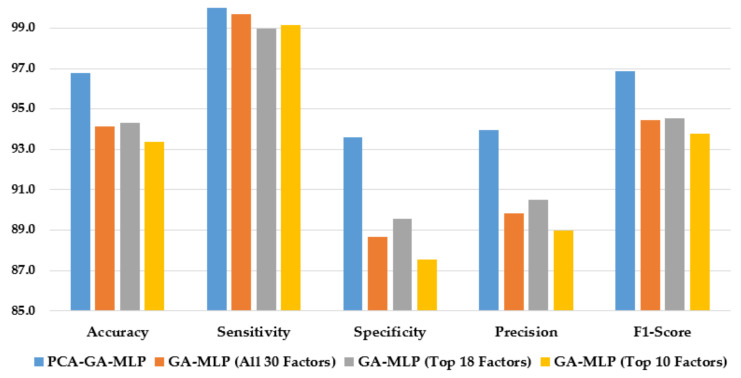
Comparison of the proposed method using four feature selection methods to diagnose Schiller.

Figure 4 illustrates the performance of the hybrid system of PCA–GA–MLP and the GA–MLPs that incorporated the top 10 factors, the top 18 factors, and all 30 factors to diagnose Schiller. The hybrid system of PCA–GA–MLP outperforms all three GA–MLPs across all performance measures (with 2–3% improvement in accuracy, 4–6% improvement in specificity, 3–5% improvement in precision, and 2–3% improvement in F1-score). The GA–MLPs that used the top 10 factors, the top 18 factors, and all 30 factors to diagnose Schiller all have similar performance, regardless of the number of factors included in the GA–MLP.

The proposed method, PCA–GA–MLP, is compared with the best approaches in the literature in the diagnosis of Schiller, using the same benchmark dataset from UCI. As shown in Table 8, PCA–GA–MLP performs better than the methods reported by Wu and Zhou [4], Abdoh et al. [22], Deng et al. [23], and Wahid and Al-Mazini [5] in terms of accuracy, sensitivity, and F1-score. The PCA–GA–MLP method achieves the highest sensitivity when compared with all other approaches. However, the method of Alsmariy et al. [24] has slightly better accuracy, specificity, and F1-score than the proposed PCA–GA–MLP method.

### 5.3. Target Variable: Cytology

Table 9 compares the performance of GA–MLPs with other algorithms in diagnosing Cytology. When implementing PCA as a dimensional reduction technique, PCA–GA–MLP achieves the highest accuracy, specificity, precision, and F1-score of 97.54%, 95.15%, 95.27%, and 97.56%, respectively. The next best method is PCA–RF, which has an accuracy of 91.58%, a sensitivity of 95.30%, a specificity of 88.06%, a precision of 88.73%, and an F1-score of 91.83%.

When using all available (30) factors, only the top 18 factors, or only the top 10 factors to diagnose Cytology, GA–MLP outperforms all other methods across all performance measures, followed by KNN and CD-KNN.

**Figure 5:**
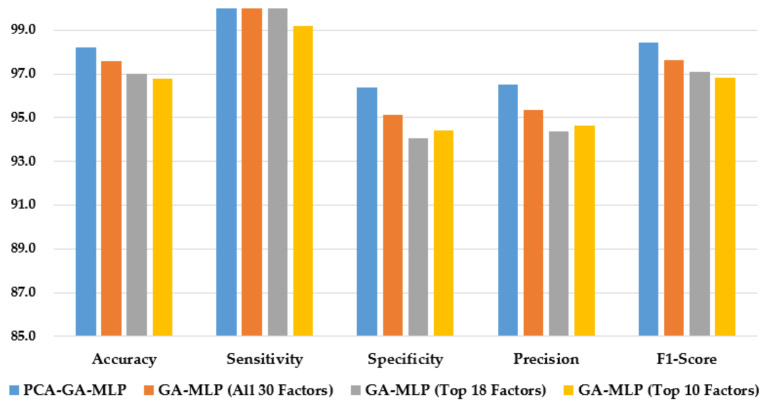
Comparison of the proposed method using four feature selection methods to diagnose Cytology.

Figure 5 compares the performance of the hybrid system of PCA–GA–MLP and the GA–MLPs that used the top 10 factors, the top 18 factors, and all 30 factors to diagnose Cytology. As shown in Figure 5, the hybrid system of PCA–GA–MLP outperforms all three GA–MLPs across all but one performance indicator. Comparing the performance of GA–MLPs that used the top 10 factors, the top 18 factors, and all 30 factors to diagnose Cytology, some improvement in model performance is observed when a larger number of factors is used in the GA–MLP.

Table 10 compares PCA–GA–MLP with other approaches from the literature in diagnosing Cytology. PCA–GA–MLP achieves better accuracy and F1-score than all reported approaches. In terms of sensitivity, PCA–GA–MLP ranks second, next to the method reported by Wu and Zhou [4]. In terms of specificity, PCA–GA–MLP performs better than the methods reported by Wu and Zhou [4] and Alsmariy et al. [24].

### 5.4. Target Variable: Biopsy

Table 11 compares the performance measures of GA–MLP with other classifiers to diagnose Biopsy. When adopting PCA as a dimensional reduction tool, PCA–GA–MLP achieves the highest accuracy, specificity, precision, and F1-score of 97.75%, 95.54%, 95.63%, and 97.76%, respectively. PCA–AdaBoost is the second-best method with an accuracy of 90.93%, a sensitivity of 94.24%, a specificity of 87.90%, a precision of 88.47%, and an F1-score of 91.19%.

When using all 30 factors or the top 10 factors to diagnose Biopsy, GA–MLP outperforms all other approaches in all performance measures. In the case of using the top 18 factors to diagnose Biopsy, GA–MLP outperforms all other approaches in terms of accuracy, sensitivity, precision, and F1-score.

**Figure 6:**
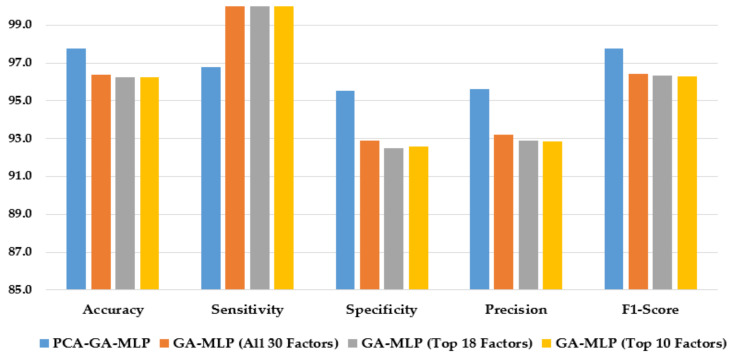
Comparison of the proposed method using four feature selection methods to diagnose Biopsy.

Figure 6 compares the performance of the hybrid system of PCA–GA–MLP, and the three GA–MLPs that used the top 10 factors, the top 18 factors, and all 30 factors to diagnose Biopsy. As shown in Figure 6, the hybrid system of PCA–GA–MLP outperforms all three GA–MLPs across all but one performance indicators. All three GA–MLPs have similar performance regardless of the number of factors included in the GA–MLP models.

Table 12 compares the performance of PCA–GA–MLP with other approaches from the literature in diagnosing Biopsy. PCA–GA–MLP achieves better accuracy and F1-score than all reported approaches. In terms of sensitivity, PCA–GA–MLP ranks third, next to the methods reported by Wu and Zhou [4] and Alsmariy et al. [24].

### 5.5. Four Target Variables

This section compares the classification accuracy of the proposed PCA–GA–MLP method with reported studies from the literature for all four target variables of cervical cancer (HSCB) on the same dataset from UCI. The classification accuracy is the only performance measure that is reported in the six relevant studies from the literature.

**Figure 7:**
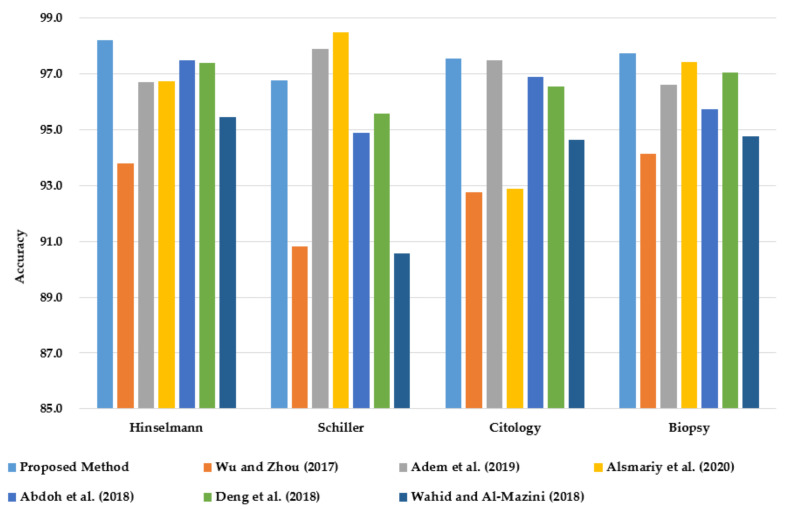
Comparison of classification accuracy of PCA–GA–MLP with the best methods in the literature in diagnosing cervical cancer using the benchmark dataset from UCI.

As shown in Figure 7, PCA–GA–MLP achieves the highest classification accuracy in diagnosing Hinselmann, Cytology, and Biopsy. In diagnosing Schiller, PCA–GA–MLP ranks third, next to the methods reported by Alsmariy et al. [24] and Adem et al. [15].

## 6. Conclusions and Future Work

This research proposed an integrated system of PCA, GA, and MLP for diagnosing cervical cancer cases using the benchmark dataset from UCI. There are four target variables of cervical cancer in the dataset: Hinselmann, Schiller, Cytology, and Biopsy. Four feature selection approaches were explored; dimensional reduction was performed using the PCA method, and different subsets of the original factors were selected based on Random Forest Importance.

Experimental results show that the hybrid system of PCA–GA–MLP outperforms all other classification algorithms and the three GA–MLP versions on all four target variables. In comparison with the existing approaches in the literature that were implemented on the same cervical cancer dataset, the PCA–GA–MLP model achieves the highest classification accuracy in Hinselmann, Cytology, and Biopsy (98.20%, 97.54%, and 97.75%, respectively).

Given the growing interest in cervical cancer research using ML algorithms, this research developed a robust predictive tool for cervical cancer. Physicians and healthcare providers can use the proposed model to identify patients with cervical cancer in its early stages. For the identified cases, the medical team can focus their effort on preventive actions and plans to improve women’s care and ultimately reduce cervical cancer rates and the associated costs.

The proposed research has a few limitations. The current approach did not consider RFE as a tool for selecting the best risk factors. Furthermore, the random oversampling technique was used to balance the unbalanced dataset. For future work, feature selection using RFE and advanced data balancing techniques such as SMOTE and cost-sensitive learning can be explored in the proposed method to further improve the overall performance.

## Figures and Tables

**Table 1:** Risk factors of cervical cancer.

Factor Number	Factor Name	Factor Number	Factor Name
1	Age	17	STDs: vulvo-perineal condylomatosis
2	Number of sexual partners	18	STDs: syphilis
3	First sexual intercourse (age)	19	STDs: pelvic inflammatory disease
4	Number of pregnancies	20	STDs: genital herpes
5	Smokes	21	STDs: molluscum contagiosum
6	Smokes (years)	22	STDs: AIDS
7	Smokes (packs/year)	23	STDs: HIV
8	Hormonal contraceptives	24	STDs: Hepatitis B
9	Hormonal contraceptives (years)	25	STDs: HPV
10	Intrauterine Device (IUD)	26	STDs: Number of diagnoses
11	IUD (years)	27	STDs: Time since first diagnosis
12	Sexually Transmitted Diseases (STDs)	28	STDs: Time since last diagnosis
13	STDs (number)	29	Dx: Cancer
14	STDs: condylomatosis	30	Dx: CIN
15	STDs: cervical condylomatosis	31	Dx: HPV
16	STDs: vaginal condylomatosis	32	Diagnosis (Dx)

**Table 2:** Top 18 factors for four target variables based on RF relative importance sequence.

Hinselmann	Schiller	Cytology	Biopsy
1	1	1	1
9	9	3	9
3	3	9	3
2	4	2	4
4	2	4	2
31	11	7	12
6	7	11	7
7	8	6	11
23	17	23	32
11	6	31	8
17	31	32	6
12	12	26	17
8	5	8	18
29	23	5	10
14	29	14	5
5	16	18	29
26	26	12	30
16	10	29	21

**Table 3:** Top 10 factors for four target variables based on RF relative importance sequence.

Hinselmann	Schiller	Cytology	Biopsy
1	1	1	1
9	9	3	9
3	3	9	3
2	4	2	4
4	2	4	2
31	11	7	12
6	7	11	7
7	8	6	11
23	17	23	32
11	6	31	8

**Table 4:** Confusion matrix.

		Predicted Class	
		Non-Cervical Cancer	Cervical Cancer
Actual Class	Non-Cervical Cancer	TN	FP
	Cervical Cancer	FN	TP

**Table 5:** Comparison of the proposed method using four feature selection methods with nine other classification algorithms in diagnosing Hinselmann.

Feature Selection: PCA/Method	Performance Measures				
	Accuracy	Sensitivity	Specificity	Precision	F1-Score
**PCA–GA–MLP**	98.20	100.00	96.37	96.54	98.24
**PCA–RF**	92.16	98.67	85.57	87.22	92.58
**PCA–LDA**	61.05	52.17	69.93	63.24	57.05
**PCA–SVM**	75.23	84.81	65.52	71.07	77.28
**PCA–LR**	64.11	60.65	67.56	64.95	62.67
**PCA–NB**	60.34	100.00	20.58	55.75	71.59
**PCA–KNN**	85.81	100.00	71.67	77.93	87.57
**PCA–DT**	83.39	95.69	71.31	76.98	85.21
**PCA–AdaBoost**	94.67	100.00	89.29	90.41	94.96
**PCA–CD-KNN**	84.40	99.55	69.29	76.53	86.49
**Feature Selection:** **All 30 Factors/Method**	**Performance Measures**				
	**Accuracy**	**Sensitivity**	**Specificity**	**Precision**	**F1** **-** **Score**
**GA–MLP**	97.57	100.00	95.15	95.36	97.62
**RF**	85.27	86.09	84.47	84.59	85.09
**LDA**	72.33	76.01	68.65	70.83	73.28
**SVM**	75.39	87.42	63.36	70.58	78.06
**LR**	72.33	75.14	69.43	71.07	72.96
**NB**	51.57	100.00	3.11	50.79	67.35
**KNN**	85.58	100.00	71.14	77.73	87.42
**DT**	71.40	95.71	47.24	64.69	77.06
**AdaBoost**	87.07	93.58	80.47	82.69	87.74
**CD-KNN**	85.66	99.39	71.90	78.07	87.42
**Feature Selection:** **Top 18 Factors/Method**	**Performance Measures**				
	**Accuracy**	**Sensitivity**	**Specificity**	**Precision**	**F1** **-** **Score**
**GA–MLP**	97.02	100.00	94.08	94.36	97.09
**RF**	85.11	88.04	82.23	83.17	85.32
**LDA**	70.92	73.24	68.46	69.91	71.48
**SVM**	71.71	84.75	58.63	67.27	74.95
**LR**	71.00	73.24	68.63	70.03	71.53
**NB**	55.25	100.00	10.39	52.76	69.07
**KNN**	85.42	100.00	70.82	77.50	87.29
**DT**	71.32	95.71	47.09	64.62	77.01
**AdaBoost**	87.07	93.58	80.47	82.69	87.74
**CD-KNN**	84.40	99.55	69.29	76.53	86.49
**Feature Selection:** **Top 10 Factors/Method**	**Performance Measures**				
	**Accuracy**	**Sensitivity**	**Specificity**	**Precision**	**F1** **-** **Score**
**GA–MLP**	96.79	99.21	94.40	94.65	96.84
**RF**	83.39	95.68	71.24	77.15	85.18
**LDA**	66.93	68.13	65.58	66.50	67.28
**SVM**	67.79	68.23	67.49	67.62	67.68
**LR**	66.46	68.15	64.66	65.97	66.95
**NB**	61.98	50.85	73.01	64.58	55.01
**KNN**	84.40	100.00	68.79	76.24	86.50
**DT**	70.61	97.25	44.04	63.62	76.83
**AdaBoost**	85.74	92.13	79.15	81.58	86.43
**CD-KNN**	83.86	99.39	68.40	75.86	86.01

**Table 6:** Comparison of the proposed method with the best results in the literature in diagnosing Hinselmann.

Study	Validation	Method	Accuracy	Sensitivity	Specificity	Precision	F1-Score
**Proposed Method**	5-Fold Cross-Validation	PCA–GA–MLP	98.20	100.00	96.37	96.54	98.24
**[4]**	5-fold cross-validation	SVM SVM–RFE SVM–PCA	93.79	100.00	89.96	N/A	N/A
**[15]**	70% training and 30% testing	Deep Learning (stacked autoencoder and softmax)	96.70	N/A	N/A	N/A	N/A
**[24]**	10-fold cross-validation	PCA, SMOTE with voting on LR, RF, DT	96.73	96.50	97.69	NA	96.85
**[22]**	10-fold cross-validation	SMOTE–RF SMOTE–RF–RFE SMOTE–RF–PCA	97.60	96.65	98.54	N/A	N/A
**[23]**	58% training and 42% testing	SMOTE with RF, SVM, and XGBoost	97.39	95.14	99.49	N/A	96.50
**[5]**	5-fold cross-validation	ACO and Ant-Miner data classification rule	95.45	N/A	N/A	N/A	N/A

**Table 7:** Comparison of the proposed method using four feature selection methods with nine other classification algorithms in diagnosing Schiller.

Feature Selection: PCA/Method	Performance Measures				
	Accuracy	Sensitivity	Specificity	Precision	F1-Score
**PCA–GA–MLP**	96.78	100.00	93.61	93.97	96.87
**PCA–RF**	86.03	81.99	90.05	89.22	85.42
**PCA–LDA**	65.04	52.38	77.61	69.92	59.74
**PCA–SVM**	70.50	54.18	86.74	80.11	64.25
**PCA–LR**	63.88	51.62	76.13	68.17	58.70
**PCA–NB**	51.32	95.35	7.32	50.73	66.20
**PCA–KNN**	76.69	94.00	59.31	69.96	80.08
**PCA–DT**	75.04	68.31	81.65	80.68	72.60
**PCA–AdaBoost**	86.53	90.27	82.84	83.98	86.98
**PCA–CD-KNN**	87.52	99.15	75.88	80.50	88.83
**Feature Selection:** **All 30 Factors/Method**	**Performance Measures**				
	**Accuracy**	**Sensitivity**	**Specificity**	**Precision**	**F1** **-** **Score**
**GA–MLP**	94.13	99.67	88.68	89.82	94.45
**RF**	71.07	54.37	87.68	81.65	65.11
**LDA**	64.71	51.08	78.25	70.13	58.98
**SVM**	69.75	50.20	89.16	82.59	62.16
**LR**	63.80	51.08	76.45	68.32	58.36
**NB**	51.16	100.00	2.33	50.59	67.18
**KNN**	75.37	93.32	57.34	68.69	79.08
**DT**	67.69	55.13	80.49	75.45	62.17
**AdaBoost**	72.64	68.42	76.82	74.58	71.29
**CD-KNN**	85.29	95.86	74.70	79.19	86.72
**Feature Selection:** **Top 18 Factors/Method**	**Performance Measures**				
	**Accuracy**	**Sensitivity**	**Specificity**	**Precision**	**F1** **-** **Score**
**GA–MLP**	94.30	98.97	89.56	90.50	94.53
**RF**	71.40	54.18	88.48	82.92	65.35
**LDA**	64.79	51.08	78.44	70.19	59.02
**SVM**	68.60	51.33	85.70	78.34	61.79
**LR**	63.47	50.93	75.94	67.79	58.06
**NB**	50.41	97.03	3.84	50.22	66.17
**KNN**	75.37	93.32	57.33	68.71	79.08
**DT**	67.60	55.13	80.32	75.27	62.10
**AdaBoost**	85.74	92.13	79.15	81.58	86.43
**CD-KNN**	85.70	96.68	74.69	79.32	87.13
**Feature Selection:** **Top 10 Factors/Method**	**Performance Measures**				
	**Accuracy**	**Sensitivity**	**Specificity**	**Precision**	**F1** **-** **Score**
**GA–MLP**	93.39	99.17	87.53	88.98	93.79
**RF**	73.06	56.29	89.85	84.67	67.49
**LDA**	62.81	52.27	73.39	66.02	58.25
**SVM**	65.87	45.68	86.08	76.78	56.96
**LR**	62.40	52.58	72.21	65.33	58.20
**NB**	61.90	45.32	78.44	67.68	54.18
**KNN**	75.29	93.98	56.50	68.41	79.14
**DT**	69.01	63.72	74.52	72.03	66.89
**AdaBoost**	71.74	69.62	73.86	72.80	71.12
**CD-KNN**	86.28	96.68	75.83	80.09	87.60

**Table 8:** Comparison of the proposed method with the best results in the literature in diagnosing Schiller.

Study	Validation	Method	Accuracy	Sensitivity	Specificity	Precision	F1-Score
**Proposed Method**	5-Fold Cross-Validation	PCA–GA–MLP	96.78	100.00	93.61	93.97	96.87
**[4]**	5-fold cross-validation	SVM SVM–RFE SVM–PCA	90.81	98.99	84.63	N/A	N/A
**[15]**	70% training and 30% testing	Deep Learning (stacked autoencoder and softmax)	97.90	N/A	N/A	N/A	N/A
**[24]**	10-fold cross-validation	PCA, SMOTE with voting on LR, RF, DT	98.49	98.60	98.60	N/A	98.37
**[22]**	10-fold cross-validation	SMOTE–RF SMOTE–RF–RFE SMOTE–RF–PCA	95.01	93.24	96.68	N/A	N/A
**[23]**	58% training and 42% testing	SMOTE with RF, SVM, and XGBoost	95.59	93.92	97.25	N/A	96.00
**[5]**	5-fold cross-validation	ACO and Ant-Miner data classification rule	90.56	N/A	N/A	N/A	N/A

**Table 9:** Comparison of the proposed method using four feature selection methods with nine other classification algorithms in diagnosing Cytology.

Feature Selection: PCA/Method	Performance Measures				
	Accuracy	Sensitivity	Specificity	Precision	F1-Score
**PCA–GA–MLP**	97.54	96.78	95.15	95.27	97.56
**PCA–RF**	91.58	95.30	88.06	88.73	91.83
**PCA–LDA**	62.04	47.56	76.89	67.69	55.24
**PCA–SVM**	71.01	64.86	77.01	73.83	69.01
**PCA–LR**	61.88	51.00	73.21	65.93	56.81
**PCA–NB**	53.53	94.93	12.18	51.99	67.10
**PCA–KNN**	83.56	100.00	67.08	75.33	85.87
**PCA–DT**	80.54	92.77	68.41	74.46	82.55
**PCA–AdaBoost**	89.83	96.25	83.50	85.36	90.41
**PCA–CD-KNN**	87.06	99.70	74.45	79.70	88.51
**Feature Selection:** **All 30 Factors/Method**	**Performance Measures**				
	**Accuracy**	**Sensitivity**	**Specificity**	**Precision**	**F1** **-** **Score**
**GA–MLP**	95.39	100.00	92.02	92.38	95.61
**RF**	74.27	66.32	82.61	79.20	71.96
**LDA**	63.62	56.07	71.10	66.06	60.47
**SVM**	65.05	60.61	69.06	68.31	62.72
**LR**	63.31	58.30	68.28	64.74	61.22
**NB**	52.90	100.00	5.74	51.52	67.94
**KNN**	83.24	100.00	66.43	74.89	85.61
**DT**	70.62	58.50	82.80	77.29	66.51
**AdaBoost**	77.05	84.33	70.07	73.65	78.45
**CD-KNN**	86.10	100.00	72.29	78.33	87.77
**Feature Selection:** **Top 18 Factors/Method**	**Performance Measures**				
	**Accuracy**	**Sensitivity**	**Specificity**	**Precision**	**F1** **-** **Score**
**GA–MLP**	94.92	100.00	89.92	90.75	95.12
**RF**	74.98	68.67	81.55	78.77	73.25
**LDA**	63.46	55.64	71.33	66.17	60.21
**SVM**	66.09	64.40	67.20	66.45	65.25
**LR**	63.15	57.27	69.08	64.99	60.68
**NB**	53.53	97.37	9.57	51.88	67.64
**KNN**	83.16	100.00	66.25	74.79	85.55
**DT**	70.54	58.50	82.63	77.17	66.46
**AdaBoost**	76.73	84.61	69.16	73.18	78.31
**CD-KNN**	85.94	100.00	71.88	78.11	87.65
**Feature Selection:** **Top 10 Factors/Method**	**Performance Measures**				
	**Accuracy**	**Sensitivity**	**Specificity**	**Precision**	**F1** **-** **Score**
**GA–MLP**	94.04	100.00	88.00	89.36	93.32
**RF**	80.06	82.03	78.12	78.78	80.30
**LDA**	60.61	55.31	66.68	62.63	58.14
**SVM**	68.79	72.79	65.08	67.58	69.95
**LR**	62.59	62.06	63.81	63.35	62.30
**NB**	55.76	77.63	34.24	54.37	63.71
**KNN**	82.76	100.00	65.57	74.34	85.24
**DT**	65.85	52.98	79.01	73.60	60.06
**AdaBoost**	74.82	80.97	68.85	72.12	76.13
**CD-KNN**	86.02	100.00	71.99	78.12	87.69

**Table 10:** Comparison of the proposed method with the best results in the literature in diagnosing Cytology.

Study	Validation	Method	Accuracy	Sensitivity	Specificity	Precision	F1-Score
**Proposed Method**	5-Fold Cross-Validation	PCA–GA–MLP	97.54	96.78	95.15	95.27	97.56
**[4]**	5-fold cross-validation	SVM SVM–RFE SVM–PCA	92.75	100.00	87.92	N/A	N/A
**[15]**	70% training and 30% testing	Deep Learning (stacked autoencoder and softmax)	97.50	N/A	N/A	N/A	N/A
**[24]**	10-fold cross-validation	PCA, SMOTE with voting on LR, RF, DT	92.89	93.12	94.59	N/A	93.35
**[22]**	10-fold cross-validation	SMOTE–RF SMOTE–RF–RFE SMOTE–RF–PCA	96.94	94.82	99.01	N/A	N/A
**[23]**	58% training and 42% testing	SMOTE with RF, SVM, and XGBoost	96.56	94.32	98.51	N/A	97.00
**[5]**	5-fold cross-validation	ACO and Ant-Miner data classification rule	94.64	N/A	N/A	N/A	N/A

**Table 11:** Comparison of the proposed method using four feature selection methods with nine other classification algorithms in diagnosing Biopsy.

Feature Selection: PCA/Method	Performance Measures				
	Accuracy	Sensitivity	Specificity	Precision	F1-Score
**PCA–GA–MLP**	97.75	96.78	95.54	95.63	97.76
**PCA–RF**	89.00	87.59	90.27	90.02	88.77
**PCA–LDA**	69.42	56.40	82.72	76.48	64.69
**PCA–SVM**	74.23	60.60	88.20	83.56	70.07
**PCA–LR**	67.82	55.73	80.26	73.84	63.20
**PCA–NB**	54.34	92.31	16.28	52.49	66.81
**PCA–KNN**	85.07	100.00	70.11	76.87	86.89
**PCA–DT**	84.75	98.50	71.10	77.33	86.51
**PCA–AdaBoost**	90.93	94.24	87.90	88.47	91.19
**PCA–CD-KNN**	88.60	100.00	77.22	81.31	89.66
**Feature Selection:** **All 30 Factors/Method**	**Performance Measures**				
	**Accuracy**	**Sensitivity**	**Specificity**	**Precision**	**F1** **-** **Score**
**GA–MLP**	96.39	100.00	92.90	93.19	96.45
**RF**	76.00	61.31	90.97	87.04	71.73
**LDA**	69.66	58.41	81.24	75.66	65.60
**SVM**	74.80	59.85	90.01	85.47	70.20
**LR**	69.98	59.41	80.86	75.60	66.27
**NB**	52.25	100.00	4.44	51.14	67.56
**KNN**	84.51	100.00	69.09	76.25	86.46
**DT**	73.11	57.51	89.35	84.84	68.05
**AdaBoost**	80.09	78.16	82.24	81.19	79.46
**CD-KNN**	89.49	100.00	79.21	82.54	90.35
**Feature Selection:** **Top 18 Factors/Method**	**Performance Measures**				
	**Accuracy**	**Sensitivity**	**Specificity**	**Precision**	**F1** **-** **Score**
**GA–MLP**	96.23	100.00	92.48	92.92	96.32
**RF**	77.69	62.99	92.58	89.35	73.74
**LDA**	69.02	57.36	81.07	75.00	64.72
**SVM**	71.11	58.97	83.76	78.18	66.95
**LR**	68.46	58.07	79.17	73.49	64.62
**NB**	51.61	99.14	4.17	50.85	67.09
**KNN**	83.87	100.00	67.77	75.52	85.99
**DT**	72.87	57.51	88.84	84.18	67.85
**AdaBoost**	81.62	81.05	82.10	81.90	81.39
**CD-KNN**	88.36	98.25	78.70	81.92	89.27
**Feature Selection:** **Top 10 Factors/Method**	**Performance Measures**				
	**Accuracy**	**Sensitivity**	**Specificity**	**Precision**	**F1** **-** **Score**
**GA–MLP**	96.23	100.00	92.59	92.86	96.28
**RF**	79.85	67.30	92.57	89.97	76.83
**LDA**	68.38	57.98	78.95	73.13	64.54
**SVM**	72.39	58.12	86.78	81.32	67.63
**LR**	68.22	58.15	78.47	72.77	64.49
**NB**	67.57	53.62	81.74	74.42	62.16
**KNN**	83.95	100.00	67.94	75.66	86.07
**DT**	73.11	61.81	83.45	81.37	69.48
**AdaBoost**	79.93	80.18	79.82	79.94	79.93
**CD-KNN**	89.97	100.00	79.95	83.17	90.78

**Table 12:** Comparison of the proposed method with the best results in the literature in diagnosing Biopsy.

Study	Validation	Method	Accuracy	Sensitivity	Specificity	Precision	F1-Score
**Proposed method**	5-fold cross-validation	PCA–GA–MLP	97.75	96.78	95.54	95.63	97.76
**[4]**	5-fold cross-validation	SVM SVM–RFE SVM–PCA	94.13	100.00	90.21	N/A	N/A
**[15]**	70% training and 30% testing	Deep Learning (stacked autoencoder and softmax)	96.60	N/A	N/A	N/A	N/A
**[24]**	10-fold cross-validation	PCA, SMOTE with voting on LR, RF, DT	97.44	97.79	98.01	N/A	97.44
**[22]**	10-fold cross-validation	SMOTE–RF SMOTE–RF–RFE SMOTE–RF–PCA	96.06	94.55	97.51	N/A	N/A
**[23]**	58% training and 42% testing	SMOTE with RF, SVM, and XGBoost	97.06	95.50	98.85	N/A	97.00
**[5]**	5-fold cross-validation	ACO and Ant-Miner data classification rule	94.76	N/A	N/A	N/A	N/A

## Data Availability

UCI machine learning repository published the cervical cancer dataset (on UCI Machine Learning Repository: Cervical cancer (Risk Factors) Data Set).
